# Minimally invasive subperiosteal pocket technique for Osia 2 system- implantation without fixation

**DOI:** 10.1007/s00405-024-08915-3

**Published:** 2024-08-24

**Authors:** Zsofia Bere, Roland Nagy, Balint Posta, Adam Perenyi, Henrik Fyrlund, Janos Jarabin, Balazs Dimak, Laszlo Rovo

**Affiliations:** 1 Department of Oto-Rhino- Laryngology and Head- Neck Surgery, University of Szeged, Szeged, Hungary; 2 Cochlear Bone Anchored Solutions, Mölnlycke, Sweden

**Keywords:** Osia 2 system, Minimally invasive surgery, pediatric Osia 2 surgery

## Abstract

The CochlearTM Osia^®^ 2 is an active transcutaneous implant designed to treat patients with different types of hearing loss. Due to its size, implantation needs appropriate practice since the necessity of extended flap creation and bone work can be an issue in some cases. The goal of our study was to determine whether fixation of the OSI200 implant was necessary for the performance of patients with conductive or mild mixed hearing loss.

The vibroacoustic performance of the Osia 2 system, with and without BI300 fixation, was evaluated through tests conducted on a head model. In addition, three patients underwent surgery using the modified minimally invasive subperiosteal pocket technique; the OSI200 implant was placed in a tight subperiosteal pocket without fixing it with the BI300 implant. To evaluate the audiological performance of the non-fixated Osia 2 system, we compared the preoperative unaided pure tone and suprathreshold testing with the Baha 5 sound processor and the non-fixated Osia 2 system aided thresholds.

Initial results indicate that omitting fixation does not significantly impair the function of the Osia 2 system. The findings of the clinical assessment support the fact that the Osia 2 system performed better than the Baha 5 system on Softband, both in pure tone and suprathreshold tests.

According to our results, we have found that utilizing the subperiosteal pocket method and implanting Osia 2 without BI300 fixation may be a viable option. This approach has shown promising results in terms of improving hearing ability with minimalization of surgery related complications.

## Introducion

Bone conductive hearing implants (BCHI) are ideal options for patients with conductive hearing loss (CHL), mixed hearing loss (MHL) or single-sided deafness (SSD) [1–3]- especially in cases, where the patient is unable to use hearing aids due to anatomical malformation of the ear canal, recurrent otorrhoea or is contraindicated for cochlear implantation [1, 3]– [7]. In the last decades BCHI has undergone significant development to improve audiological performance and streamline the implantation technique with good aesthetic outcome and low rates of postoperative complications [8–10]. In terms of pediatric application these points are even more important due to the increasing requirements and the advantages associated with early restoration of hearing abilities. Consequently, surgery of young patients needs uncomplicated procedures and safe high-capacity implants.

The Osia 2 System is an active osseointegrated steady-state implant (OSI200) hearing solution that uses digital piezoelectric stimulation. It utilizes unique Piezo Power™ transducer technology and a digital link that transfers power and data between the sound processor and implant. The Piezo Power transducer delivers powerful and consistent sound output, offering several benefits, including high-frequency sensitivity essential for speech interpretation, particularly in noisy environments. The Osia System features a slim, discreet sound processor that supports direct streaming and connectivity with various wireless devices. Since its release, the device has demonstrated a reliability rate of 99.92%. Furthermore, accelerated lifetime testing has confirmed the Piezo Power transducer’s consistent performance over time (www.cochlear.com). While the audiological benefit of the system is well established [11–13] precise preoperative planning and the use of specialized instruments are necessary for the meticulous placement of the implant. The OSI200 requires sufficient space and must be securely attached to the BI300 titanium implant, which serves as the base for the OSI200. Consequently, an extensive surgical procedure is required. In order to minimize the tension on the suture line resulting from the transducer, it is recommended to position the incision at a distance of approximately 1.5 cm from the borders of the transducer, which may provide challenges in patients with limited space such as in young children. Severe craniofacial malformation cases need preoperative cranial CT scan to determine the ideal size (3–4 mm) for the BI300 implant. The success of the titanium implant fixation and osseointegration depends on the thickness and density of the bone. In addition, the manipulation of the bone might potentially lead to problems, such as bleeding or dura exposure. Currently the number of perioperative complications with the Osia system is relatively low; the prevalence of patient injury reported for Osia identified only 31 patient injuries with over 1500 implantations in just over two years (2.1%) [14].

In order to minimize potential difficulties associated with Osia 2 surgery, a Minimally invasive Subperiosteal Pocket Technique (MSPT) was utilized in specific cases. Instead of the recommended surgical approach, OSI200 was implanted into a tight subperiosteal pocket without BI300 implantation and fixation. The technique aims to minimize the surgical approach, preserve the retroauricular vascular blood supply and reduce the risk of postoperative complications related to soft tissue. The minimal incision approach also reduces scarring in the temporal region, which is advantageous for future ear reconstruction procedures. In addition, excluding the BI300 implantation and further bonework considerably decreases the likelihood of intraoperative problems, shortens the surgical process and reduces the required equipment. Prior to any human application, vibroacoustic measurements were conducted on a head model to assess the audiologial performance of the Osia 2 system without BI300 implantation. The audiological performance of three consecutive patients was examined to demonstrate that direct bone contact with the active transducer surface can provide good auditory outcomes.

## Materials and methods

### Preoperative measurements with head simulator

**Fig. 1:**
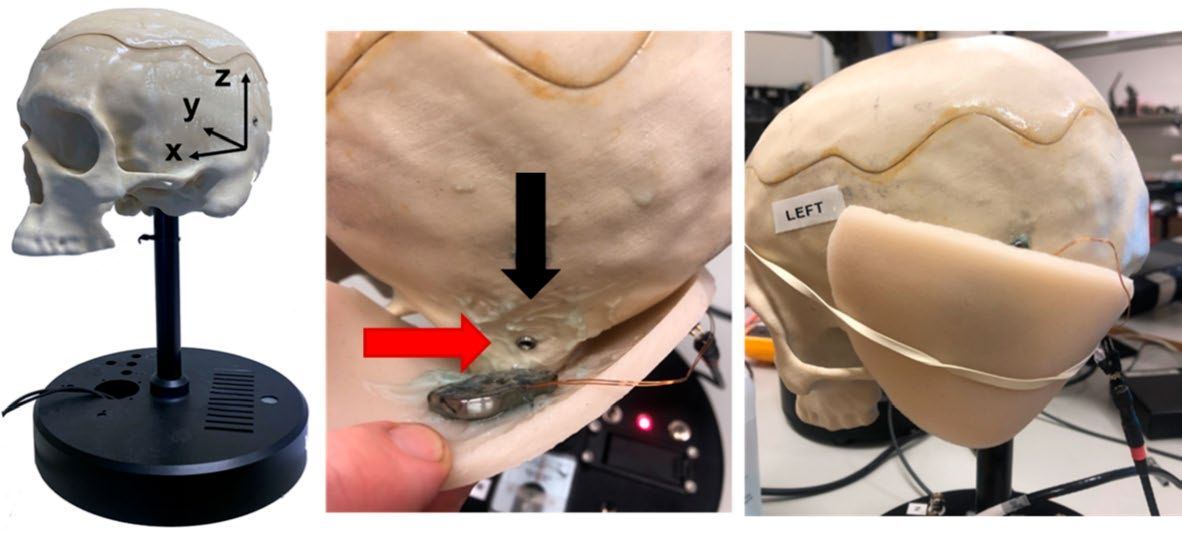
Head simulation model used to assess the output from the Osia system. The accelerometers track the propagation of the stimulus in three spatial dimensions. The magnitude of the stimulus reaching the cochlea is calculated by analysing the stimulus vectors propagating in the coronal (**z**), sagittal (**x**) and transverse/horizontal (**y**) plane. The red arrow indicates the BI300 position for the measurements anchoring the OSI200 implant to the BI300. The black arrow points to the position of the transducer in the non-BI300 fixed scenario. The transducer is coated with gel and firmly placed into the head simulator using artificial skin. The transducer was driven electrically with a 0.5 V sweep across frequencies from 100 to 20.000 Hz

To evaluate the efficiency of the OSI implant without anchoring it to the BI300 implant, comparisons of the vibroacoustic performance of Osia 2 with and without fixation was evaluated using an anatomically accurate head simulator model (Cochlear Bone Anchored Solutions, Mölnlycke) validated in cadaver studies. The model is based on a plastic skull equipped with accelerometers in the cochlea position measuring the output in the coronal, sagittal and transverse/horizontal plane. Tests with the skull simulator model were conducted comparing two scenarios; placing the Osia implant with no fixation at the temporal bone surface with gel between implant and bone, held to the surface by the artificial skin, and the normal method of anchoring the implant to the skull with a BI300 implant in the recommended implant position for Osia surgery, 50 mm posterior of the ear canal. The implant was covered by artificial skin in both test conditions (Fig. 1). A sine sweep from 100 to 20 kHz was electrically fed to the actuator. Maximum output of the OSI200 implant was measured in the non-anchored and BI300 anchored scenario in the three plane (x, y,z), bilaterally (left and right side of the head) and the results were averaged.

### Human application

The study was approved by the Hungarian Medical Research Council (ETT TUKEB: BM = 23657-1/2023)., and all individual participants provided informed consent to be included in the study.

### Candidates for surgery

#### Adult case

A 58 year old female patient with bilateral near symmetrical MHL applied for modified Osia 2 surgery. The difference in the BC threshold between the left and right sides at 500, 1000, 2000, and 4000 Hz did not exceed 10 dB. Prior attempts at rehabilitation with traditional hearing aids were unsuccessful due to recurring otorrhoea. The patient received information regarding different treatment options: Baha sound processor (SP) on Soundarc, Baha Connect, Baha Attract, Osia 2 with classic surgical procedure, and Osia 2 with MSPT. Prior to finalizing her decision, she carried out a two-week test trial of Baha 5 SP on Softband. Upon acquiring appropriate information, the patient made the decision to opt for Osia 2 with MSPT. However, it was stipulated that the BI300 implantation and system fixation would be carried out as a second stage if deemed necessary.

#### Pediatric cases

Two 6-year-old patients (one male and one female) with craniofacial malformation, additional bilateral complete ear canal atresia, and consequent bilateral symmetrical CHL were enrolled. Both patients were rehabilitated for at least 5 years with Baha 5 SP on Softband (male: unilaterally, female bilaterally), therefore they were experienced Baha users. The male patient (Pediatric 1) had previously undergone a bicoronal craniotomy in the temporal region, and preoperative CT scan revealed that the thickness of the bone in the implant position was less than 2 mm, while in case of the female patient (Pediatric 2), ear reconstruction surgery was scheduled, therefore MSPT surgery was promoted for the parents in both cases.

The fitting of the SPs was carried out with Cochlear Baha Fitting Software 6 (6.1.10625.7 version) and Osia Fitting Software 2.1.2.

**Fig. 2:**
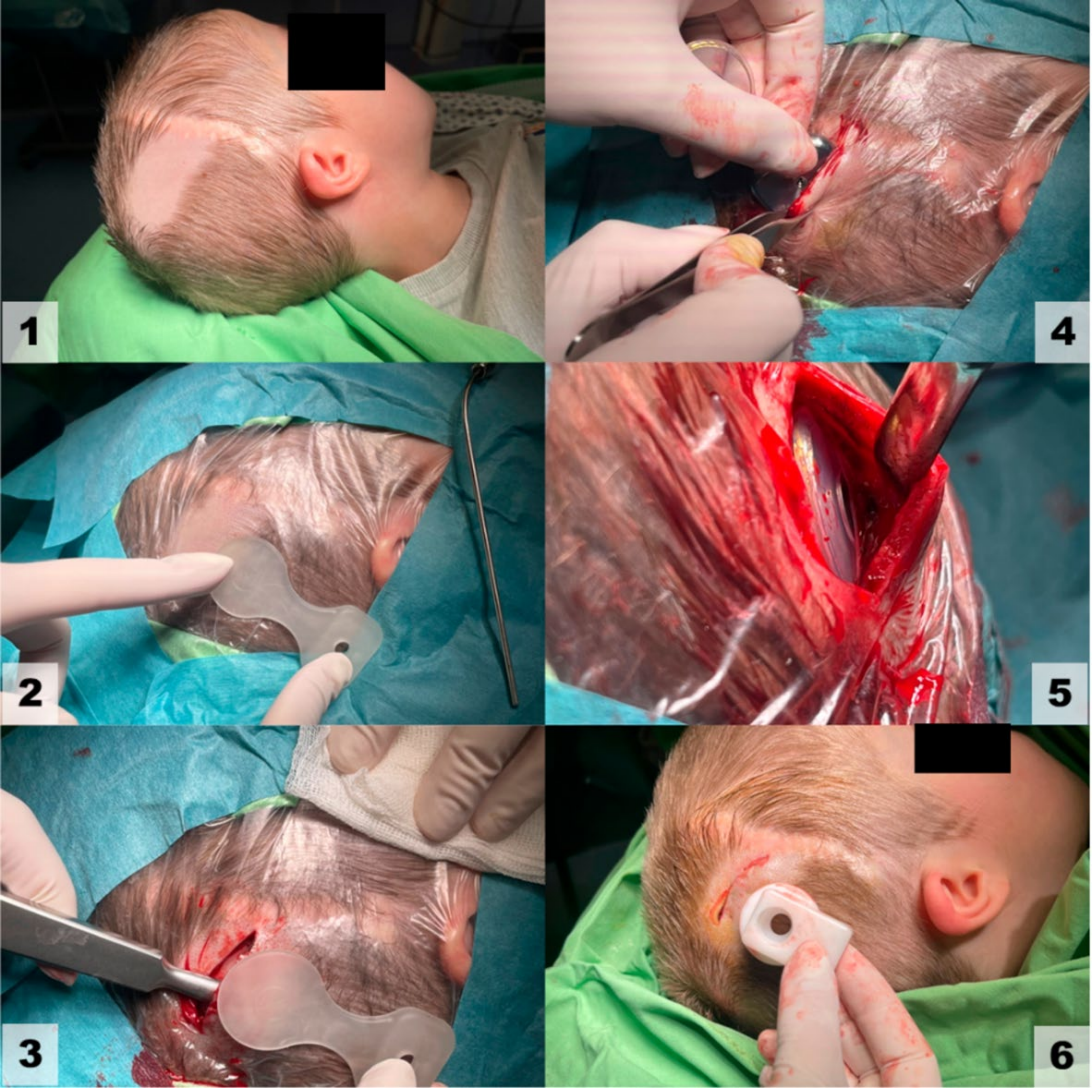
Steps of the MSPT in Osia 2 implantation. Pediatric case, 6-year-old male with bilateral ear canal atresia. (1) scar from previous craniotomy (2) implant position planning (3) elevation of the soft tissue with the periosteum from the temporal bone with Langenbeck periosteal elevator (4) insertion of the OSI200 (5) OSI200 in position (6) closure and estimated position of the sound processor

Figure 2 depicts MSPT surgery without fixation. After planning the OSI200 position, a superior temporal incision was made and a tight subperiosteal pocket was created. Compared to the manufacturer’s specifications, the transducer midline was 1.5 cm above the ear canal midline, called the tegmen position in our earlier work [15], where the temporal bone is flat. Rotating the system axis anteriorly prevented the Osia SP from being positioned too high. After two layers of wound closure, compression packing was used. On the first postoperative day, patients were emitted with compression headbands. After one week, wound healing was controlled.

### Pre and postoperative audiological measurement

Unaided and aided pure tone audiometry (PTA) and speech reception threshold (SRT) in quiet measurement was performed on each individual. Unaided air conduction (AC) thresholds with supra-aural headphone and bone conduction (BC) thresholds with bone oscillator over the mastoid process were measured at 250, 500, 1000, 2000, 4000 and 8000 Hz in audiometric booth. Non-tested ear was masked with narrow band noise via headphone. Preoperatively, aided pure tone threshold measurement on the same test frequencies was carried out in free field using the Baha 5 SP attached to Softband and postoperatively at first fitting (6th postoperative week) with Osia 2 SP. Speech tests were conducted in accordance with recommended protocols using Götze’s Hungarian Speech Test (Surján L., 1975) [16]. We determined the SRT of the three implantees as the softest level (dB HL) at which the patient accurately repeated phonetically balanced responses 50% of the time. As the Pediatric 2 patient had previously fitted bilaterally, the preoperative aided threshold testing (PTA, SRT) proceeded by using a single SP on the implant site and subsequently using SPs attached bilaterally to her Softband.

In addition, for the adult patients, a word recognition in noise test was conducted to assess the speech reception threshold in noise (SRT50) using a speech-front, noise-front (S0N0) setup, with the speaker located one meter away from the patient. The SRT50 values were assessed under three conditions: preoperatively without SP than with the Baha 5 SP on a Softband, and postoperatively with the Osia system. The measurements were taken using a fixed speech-shaped noise at 65 dB SPL to determine the level at which 50% accuracy was achieved. Initially, both the background noise and speech level were set to 65 dB SPL with a signal-to-noise ratio of 0. Subsequently, the speech level was adjusted according to the participant’s accuracy, either lowering or rising first by 5 dB and then by 2 dB.

The data were analyzed using GraphPad Prism Software (Version 10.1.2, Boston, United States). Due to the low number of cases, a descriptive analysis of the data was conducted.

## Results

### Preoperative measurements with head simulator

To express velocity (m/s/V) in dB, the following formula was applied:

$$dB = 20 \times log10\left( {{{0.01m/s/V} \over {1m/s/V}}} \right)$$

$$\:dB=20xlog10\left({10}^{-2}\right)$$

$$\:dB=20xlog10(-2)$$

$$\:dB=20x-2$$

$$\:dB=-40$$

Which indicates that, 0.01 m/s/V is eqivalent to -40dB relative to 1 m/s/V.

**Fig. 3:**
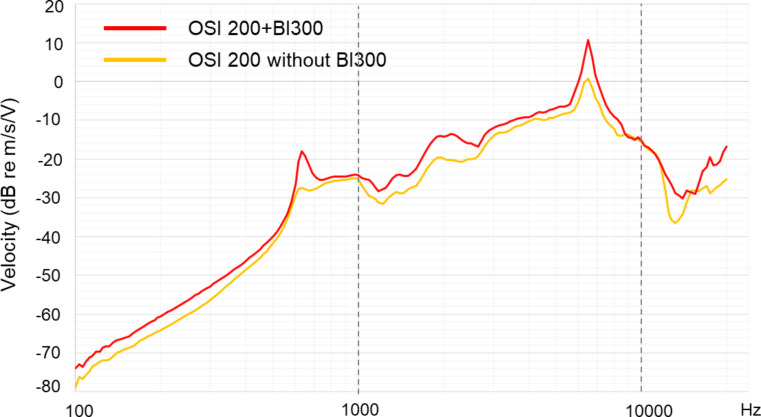
Transmission efficiency of the OSI200 implant anchored to the BI300 and covered by artificial skin (red curve) and placed on the temporal bone surface with gel under the implant covered by artificial skin (yellow curve). Velocity was better with OSI200 fixed to the BI300 implant in almost all frequencies. Average difference along the test frequencies is 2.1 dB

Figure 3 shows transmission efficiency of the OSI200 under BI300 fixed and non-fixed scenarios. Logarithmic maximum summation of the vectors represents vibration in the position of the cochlea showed that the Osia implant yielded greater output when fixed with the BI300 implant by an average of 2.1 dB difference across frequencies from 100 to 10 kHz. Although the overall variation in transmission efficiency is small, it was observed that in speech frequencies, the transmission efficiency difference ranged from 5 to 8 dB in favor of the OSI200 device connected to the BI300 implant

### Human application

Surgery was performed under local anesthesia in the adult case and under general anesthesia in the two pediatric patients. The surgery required 8 ± 1.5 min from incision to wound closure. Soft tissue thickness at SP level was ≤ 9 mm, hence no soft tissue reduction was needed. No wound healing complications occurred during the first week of control or at later visits. Based on the patient’s feedback the minimal protrusion of the OSI200 did not result in any cosmetic side effects or discomfort, despite that no bone bed was created for the transducer. After surgery (9 months after adult implantation, 4 months after Pediatric 1 and 2 months after Pediatric 2 implantation), no implant migration was found. All patients expressed satisfaction with both the aesthetic and audiological outcomes. All three patients reported better performance with the non-fixed Osia device compared to both the unaided condition and the Baha 5 Softband-aided condition. Additionally, the family reported improved speech comprehension; the children listened to the television more quietly than when using their own Baha Softband with Baha 5 SP; and the teacher reported a significant improvement in the performance of the Pediatric 2 child in dictation tasks.

**Fig. 4:**
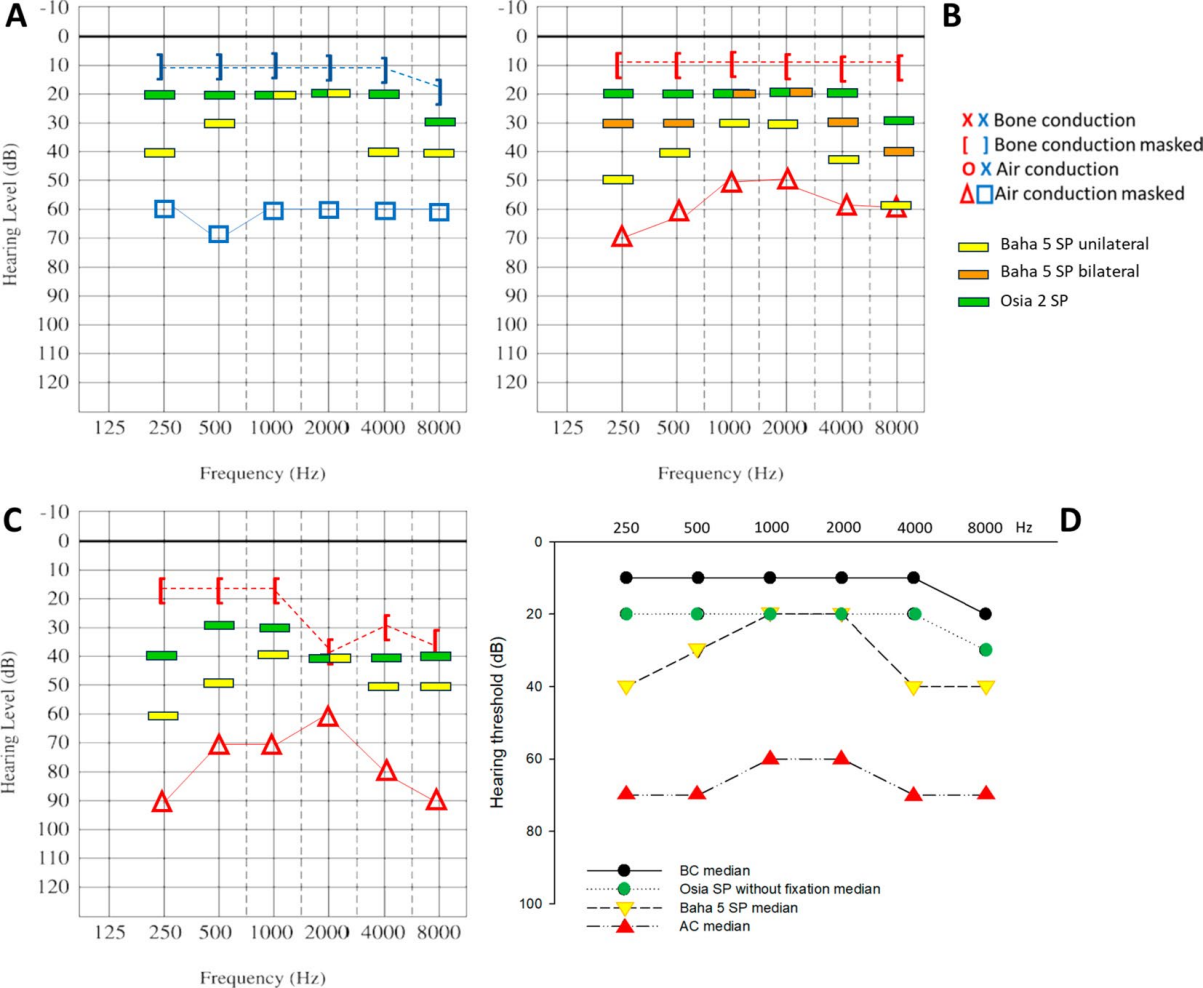
Pure tone audiometry (PTA) results of the three patients. Unaided air conduction (AC) thresholds were assessed with supra-aural headphone, while bone conduction (BC) thresholds were determined with bone oscillator above the mastoid. Non-tested ear was masked with narrow band noise via headphone. Measurements were performed in audiometric booth. Aided threshold with Baha 5 and Osia 2 (MSPT) were measured in free field. Figure 4/A Pediatric 1; Fig. 4/B Pediatric 2; Fig. 4/C Adult patient. Figure 4D unaided AC, BC and aided threshold medians. Site of Osia implantation: Adult-right side; Pediatric 1 (6 years old male)- left side; Pediatric 2 (6 years old female)- right side. Yellow mark: unilateral Baha 5 SP on Softband; orange mark: bilateral Baha 5 SP on Softband (only Pediatric 2); green mark: Osia 2 (MSPT) without BI300 fixation

Figure 4 displays unaided and aided pure tone thresholds with the Baha 5 SP on Softband and Osia 2 systems after MSPT without fixation. Both systems provide significant hearing improvement compared to the unaided situation, and Osia outperformed Baha 5 SP in each case. Adult and Pediatric 1 case were aided preoperatively with unilateral Baha 5 Softband. Preoperative aided thresholds were tested in the Pediatric 2 patient’s case (Fig. 4/B) using bilateral Baha 5 SP on Softband, following her initial bilateral rehabilitation five years ago. However, we also performed a unilateral Baha 5 Softband test at the implantation site and presented the results on the diagram (orange mark).

The audiological improvement in each test frequency (AC minus aided threshold) was most pronounced in Osia 2’s performance without fixation, especially in the high and low frequencies. Although, both the Baha 5 SP on Softband and the Osia 2 without fixation showed substantial improvement (Table 1).

**Table 1:** Audiological improvement on the PTA test frequencies with Baha 5 SP on Softband and Osia2 without fixation. Improvement calculated as AC-aided threshold. Median values of each frequencies are presented. As the Pediatric 2 patient had previously undergone rehabilitation with bilateral Baha SP, the functional gain was determined using the data obtained with bilateral Baha 5 SP in her case

Improvement (dB)	250	500	1000	2000	4000	8000
Baha 5 SP on Softband	40	30	20	20	40	40
Osia 2 without fixation	20	20	20	20	20	30
Difference in functional gain	20	10	0	0	20	10

SRT measurement showed notable improvement with both systems compared to the unaided situation, however, Osia 2 provided further improvement compared to the Baha 5 SP. The median unaided SRT was 65 dB HL, while the median aided thresholds were 40 dB HL with the Baha 5 SP and 20 dB HL with the Osia, respectively (Table 2). Since the Pediatric 2 patient was adapted to bilateral Baha 5 SP on Softband, Table 2 shows SRT with Baha 5 SP results. In her case, SRT with the unilateral Baha Softband test was 50 dB.

**Table 2:** Speech reception threshold in quiet of the individuals implanted with MSPT without fixation. Data are presented in dB HL. Pediatric 1 Baha 5 threshold: unilateral softband; Pediatric 2 Baha 5 thresholds: bilateral Softband

	Unaided	Baha 5	Osia
Adult	70	50	35
Pediatric 1	60	40	20
Pediatric 2	65	30	20
Mean	65	40.0	25
SD	5	10.0	8.7

**Fig. 5:**
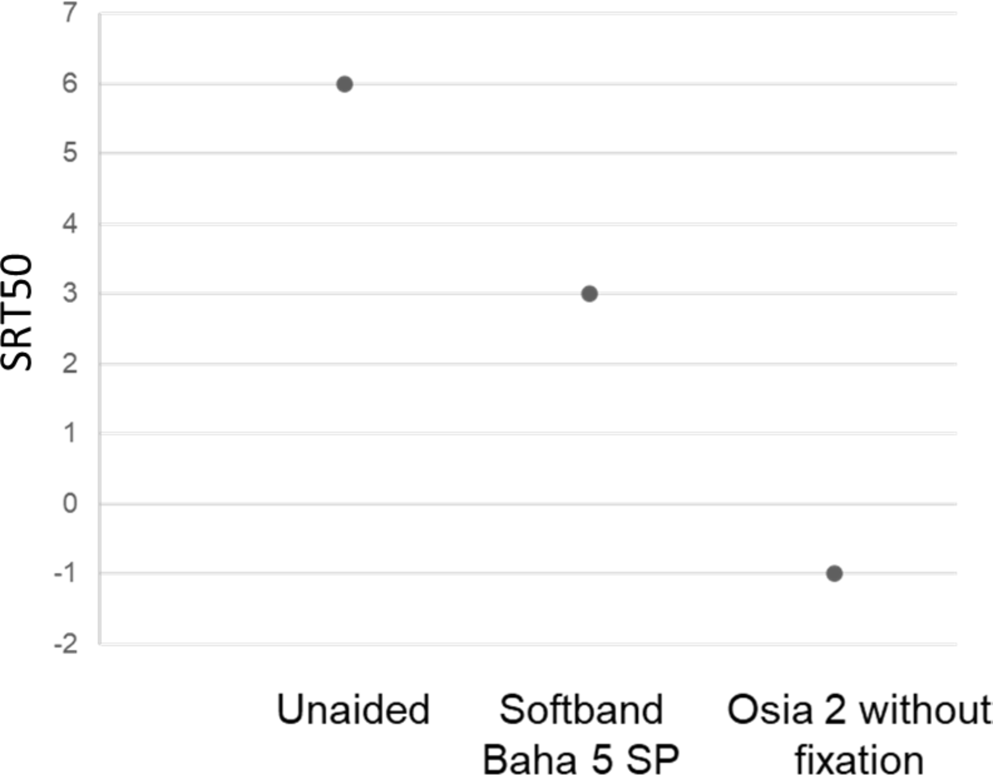
Speech reception threshold in noise (S0N0) in the unaided situation, with the Baha 5 SP on Softband and Osia 2 with MSPT without fixation of the transducer

Figure 5 represents speech reception threshold in noise of the adult patient preoperatively (unaided and with Baha 5SP on Softband) and postoperatively with the non-fixed Osia system. Both aided scores offered ≥ 3 dB improvement of SRT50 which is a considerable from a clinical perspective, however, Osia 2 with MSPT without fixation outperformed Baha 5SP on Softband demonstrating > 6dB improvement

## Discussion

When compared to standard otosurgeries, BCHI operations are simpler procedures from a technical point of view. However, it is crucial to have a thorough understanding and competence in each phase of the process. Due to the monolithic design of the Osia 2 system, the optimal positioning of the SP is directly linked to the placement of the BI300 implant. Therefore, preoperative planning of the BI300 position and OSI200 placement is an essential step and the procedure requires space for visualisation, especially for BI300 implantation and fixation of the OSI200. Although the learning curve of Osia surgery is short [11, 17], the procedure can be complicated and have a greater incidence of postoperative complications even for experienced surgeons due to the transducer size, bone thickness, or bone density issues in some cases. As per the manufacturer’s instructions, the incision line should be placed at a significant distance from the transducer borders to reduce tension on the suture line. Recommended C-shape, “lazy S”-shape, or J-shape incisions around the transducer leave long scars in the retroauricular space and usually bypass the transducer in the inferior region of the temporal area, where the risk of injury to the main regional arteries that provide blood supply to the soft tissue is high [18]. In addition, particularly in young children with underdeveloped mastoid processes, the lower section of the incision may be located too close to the neck muscles. A decrease in blood flow in this area jeopardise tissue perfusion and oxygenation, while increasing the likelihood of wound complications [21]. According to the Manufacturer and User Device Facility Experience (MAUDE) database, the most commonly reported problems with the Osia system are infection, pain, and extrusion of the device, which are predominantly treated conservatively, and only a few cases require surgical revision [14]. Previous studies on passive transcutaneous BCHI showed similar injury profiles, including discomfort, pressure injury, and wound formation, with subsequent infection or skin complications, though the majority of patients have good outcomes with these devices [14, 19]– [21]. Strong magnet compression contributes to some of these complications, while conservative treatment and magnet strength reduction typically resolve periimplant pain and erythema; in severe individual cases, skin necrosis necessitates system removal or conversion [20–22]. In contrast, active BCHIs needs much less magnetic strength as vibrations do not need to be transfered from an external source, but larger surgical approach is necessary due to their relatively big dimension compared to a passive percutaneous or transcutaneous system. An extended approach with large flap creation, particularly when the incision line crosses the proximal section of the primary arterial supply of the temporal region, might result in excessive bleeding and decreased perfusion of soft tissues, leading to difficulties in wound healing [18].

This observation is also supported by cochlear implantation (CI) studies, that described a correlation between large incisions, extended surgical approaches, and soft tissue complications such wound infections, seromas, and hematomas indicating that a reduced surgical approach minimised the occurrence of significant skin problems [23, 24]. Additionally in malformation cases, the presence of excessive scar tissue and impaired blood circulation is undesirable when ear reconstructive surgery is planned in the future. Using a different surgical approach above the transducer or between the coil-transducer area can decrease the length of the incision, reduce the size of the scar in the temporal region, and improve visibility for BI300 implantation [25–27]. However, there may still be difficulties in successfully implanting the BI300 if the bone density or thickness is inadequate. Our earlier morphometric study on the pediatric population revealed that the bone thickness in the age range of 5–6 years is around 3.5 mm at the BI300 level. Therefore, it is safe to use a 3 mm titanium implant [15]. Nevertheless, there is a growing necessity to lower the age restriction for surgery, considering the favorable audiological findings that have been reported by several groups [11, 12, 28]– [30]. Determining the bone thickness using CT is necessary for planning and safety reasons in much younger individuals or in cases of severe craniofacial malformations. In addition to irradiating the skull, the cooperation of youngsters can be a challenge and necessitates anesthesia, which raises the likelihood of complications and the overall expense of rehabilitation. The biggest advantage of our minimal invasive technique is that the incision is in the furthest possible position from the transducer, and no BI300 implantation is needed. The necessary incision length is the width of the transducer (3.5–4 cm) at the superior region of the temporal area, which leaves the retroauricular region scar-free and preserves the blood supply of the region. Due to the precise matching of the subperiosteal pocket and the OSI200 size, the flap preparation is minimal. Furthermore, the tight pocket securely holds the transducer in place and facilitates optimal contact with the bone surface which allows effective signal transmission even without the BI300, as evidenced by the vibroacoustic measurements as well as by audiological findings. Although emissary veins can lead to difficulties, the risk can be reduced by utilizing a preoperative CT scan and/or performing soft tissue flap elevation and bleeding control with the use of an endoscope. If bleeding cannot be managed, the endoscopic method can also assist in identifying the direction and required size of the further incision.

However, the duration of the follow-up in this study is limited, there is no evidence of any migration of the implant. Based on earlier research on CI, when the receiver-stimulator is placed in a tightly secured subperiosteal pocket without fixation, the likelihood of subsequent migration is minimal [31–34]. Remodelling and spontaneous development of bone surrounding the receiver-stimulator have also been described in these cases [31], which presumably will occur with the MSPT approach as well. Additionally, BI300 implantation and fixation of OSI200 is still possible later. The technique’s MRI compatibility raises questions; however, subperiosteally implanted non-fixed CI receiver-stimulators are also MRI compatible (for Nucleus 600 portfolio to 3Tesla) with an MRI kit or tight bandage, even with a magnet in its place. Therefore, MRI measurements might be non-problematic in compliance with the recommended safety rules (www.cochlear.com). In addition, it is important to note that the transducer is non-magnetic. Therefore, if removal is required, only the Osia magnet should be taken out. The entire OSI200 implant may be readily removed, even without the need for any specialized equipment, if deemed essential.

Prior research of Dolhen et al. on the Baha Attract system demonstrated that inserting the Attract internal magnet (BIM400 internal magnet) into a tight subperiosteal pocket without any fixation did not have a detrimental impact on the audiological results. In terms of PTA and SRT, the non-fixed Baha Attract yielded superior outcomes compared to the unaided or Baha 5 SP aided (on Soundarc) situations. Moreover, this minimally invasive pocket technique provided short surgical time, reduced incision, fast and uncomplicated wound healing with good esthetical outcome [35]. In our study, vibroacoustic measurements on head model also indicated, that omitting the fixation of the Osia system to BI300 do not substantially reduce the signal transmission in the level of the cochlea compared to the fixed scenario. The findings are further corroborated by our case series, as both PTA and suprathreshold testing shown a substantial improvement after the surgery using the non-fixated Osia system, moreover, in the case of the previously bilaterally rehabilitated atresia patient, the unilateral, non-fixed Osia system provided better audiological results than the bilateral Baha 5 SP on Softband, which was also supported by the subjective feedback of the patient and the family.

The Baha 5 SP on Softband demonstrated a significant change in PTA, with a threshold decrease of about ~ 25–30 dB, which aligns with the results of previous research [22, 35]. Furthermore, the Osia 2 without fixation showed an even higher improvement, with an additional threshold reduction of around 9 dB. When comparing the PTA results, it was seen that improvement of high and low frequency thresholds was more noticeable with the non-fixed Osia 2 system. Additionally, the greatest difference in average aided thresholds between adjacent frequencies did not exceed 5 dB, while in the case of the Baha 5 SP system, this difference reached 10 dB.

Previous studies have confirmed that improved performance at high frequencies enhances speech recognition and optimizes the identification and distinction of phonemes with high frequency energy [36–38]. However, insufficient high-frequency gain and inadequate maximum power output (MPO) can lead to distortion and feedback, which can negatively impact the BCHI performance, particularly in noisy environments. Our suprathreshold test results demonstrates, that MPO of the non-fixed Osia 2 system provides effective amplification, even in noise.

## Conclusion

Implanting the Osia 2 system without the BI300 yields favorable audiological outcomes. The results obtained from our study, which used both a head model and human patients, consistently demonstrate that the performance of the system is excellent even with omission of OSI200 fixation. The MSPT procedure we use for Osia implantation is minimally invasive, since it only involves minor incisions and minimum flap creation. Furthermore, the approach does not need any bonework. The procedure is considerably briefer than any of the already documented techniques and does not require any specialized instruments. Although, we do not recommend to perform Osia surgery without its own instrumentation as backup. Due to the MSPT procedure, which overcomes the challenging aspects of traditional Osia surgery, even the implantation of small children is now feasible. If deemed required, the BI300 can be implanted and fixed in a subsequent stage.

### Limitation of the study

Given the limited number of participants it was not possible to establish a statistically significant difference between the Baha 5 SP on Softband and Osia 2 system implanted with MSPT without BI300 fixation. However, based on clinical observations and the patient’s feedback, non-fixed Osia 2 system consistently outperformed the other SP in all conditions. In order to have a comprehensive understanding of the precise mechanism underlying enhanced audiological performance, it is necessary to conduct additional experiments with a larger number of participants. However, vibroacoustic tests were performed on the head model comparing the effect of anchoring and non-anchoring OSI200, further investigations are required to ascertain the audiological implications of excluding the fixation of the Osia system.
